# A Novel Totivirus Naturally Occurring in Two Different Fungal Genera

**DOI:** 10.3389/fmicb.2019.02318

**Published:** 2019-10-11

**Authors:** Mahmoud E. Khalifa, Robin M. MacDiarmid

**Affiliations:** ^1^The New Zealand Institute for Plant and Food Research Limited, Auckland, New Zealand; ^2^Botany and Microbiology Department, Faculty of Science, Damietta University, Damietta, Egypt; ^3^School of Biological Sciences, The University of Auckland, Auckland, New Zealand

**Keywords:** *Totivirus*, mycovirus, dsRNA, *Trichoderma*, *Clonostachys*

## Abstract

Mycoviruses are widely distributed across different phyla of the fungal kingdom. Viruses that share significant sequence similarities have been reported in different fungi, suggesting descent from a common ancestor. In this study, two fungal genera isolated from the same sample, *Trichoderma koningiopsis* isolate Mg10 and *Clonostachys rosea* isolate Mg06, were reported to have identical double-stranded RNA (dsRNA) profiles that consist of two virus-like, dsRNA elements (dsRNA-L and dsRNA-S). The complete sequence and genome organization of dsRNA-L from isolate Mg10 was determined. It is 4712 nucleotides (nt) long and contains two non-overlapping open reading frames (ORFs) that code for proteins with similarities to totiviruses. Consequently the virus was given the proposed name Trichoderma koningiopsis totivirus 1 (TkTV1/Mg10). The TkTV1/Mg10 genome structure resembles that of yeast totiviruses in which the region preceding the stop codon of ORF1 contains the elements required for -1 ribosomal frameshifting which may induce the expression of an ORF1–ORF2 (CP-RdRp) fusion protein. Sequence analyses of viral dsRNA-L from *C. rosea* isolate Mg06 revealed that it is nearly identical with that of TkTV1/Mg10. This relatedness was confirmed by northern blot hybridization and indicates very recent natural horizontal transmission of this virus between unrelated fungi. TkTV1 purified isometric virions were ∼38–40 nm in diameter and were able to transfect *T. koningiopsis* and *C. rosea* protoplasts. This is another report of a mycovirus present naturally in two taxonomically distinct fungi.

## Introduction

Mycoviruses, viruses that infect fungi, are widely distributed and have been reported in several fungal groups (Pearson et al., 2009). Mycoviruses mostly induce symptomless infections but several examples have been reported to induce notable effects on their hosts including reduced or enhanced virulence (Nuss and Koltin, 1990; Ghabrial, 1994; Lau et al., 2018). Three genome types, double-stranded RNA (dsRNA), single-stranded RNA (ssRNA), and single-stranded DNA (ssDNA), have been reported for mycoviruses; dsRNA genomes are the most frequently reported. One of the dsRNA virus families is the *Totiviridae* which accommodates members with non-enveloped isometric virions harboring a single linear dsRNA genome of 4.6–7.0 kbp in size. The genome consists of two overlapping open reading frames (ORFs) coding for a coat protein (CP or Gag) and an RNA-dependent RNA polymerase (RdRp or Pol) that could be expressed as a gag-pol fusion protein (Dinman et al., 1991; Ghabrial, 2008). Ghabrial and Suzuki (2009) have summarized at least three RdRP-expression strategies in *Totiviridae* members including: (a) as a CP/RdRP fusion protein by utilizing a ribosomal frameshifting mechanism; (b) as a CP/RdRp fusion protein, that is later released by proteolysis, without the involvement of ribosomal frameshifting; and (c) as a separate protein by utilizing a stop/restart mechanism. The family is currently classified into five genera: *Totivirus*, *Victorivirus*, *Giardiavirus*, *Trichomonasvirus*, and *Leishmaniavirus*. Only the *Totivirus* and *Victorivirus* genera have species that infect fungi; the remaining three genera include members that infect protozoa (Goodman et al., 2011; Wickner et al., 2012).

Members of the *Totivirus* genus are characterized by virions that are 40 nm in diameter, containing the linear dsRNA genome typical of their family (4.6–6.7 kbp). Some members are considered as helper viruses by providing capsids required for the separate encapsidation of associated satellite dsRNAs encoding killer proteins (Wickner et al., 2012). Novel *Totivirus* species are defined based on biological and molecular criteria. Viruses found in distinct hosts are different species, and viruses that share less than 50% of amino acid (aa) sequence identity are also considered different species (Wickner et al., 2012).

*Trichoderma koningiopsis* and *Clonostachys rosea* are both ascomycetes with biological control potential against several pathogens. *T. koningiopsis* and *C. rosea* are classified within the same order *Hypocreales* but belong to *Bionectriaceae* and *Hypocreaceae* families, respectively (Karlsson et al., 2015). *T. koningiopsis* is common and a cosmopolitan species that is known to have the ability to control several fungal pathogens, including *Thielaviopsis basicola* and *Fusarium verticillioides* (Samuels et al., 2006). Similarly, *C. rosea* is a mycoparasite that is commonly found in soil and considered a highly efficient antagonistic fungus against a wide range of pathogenic fungi, including *Botrytis cinerea* (Sutton et al., 1997; Wu et al., 2014). Poorly characterized dsRNAs have been previously reported in two isolates of *T. koningiopsis* (Yun et al., 2016) and nothing is yet known about the association of *C. rosea* with mycoviruses. In this study, we report the molecular characteristics of a novel totivirus naturally occurring in two different fungal hosts, *T. koningiopsis* and *C. rosea*.

## Materials and Methods

### Fungal Isolates and Culturing Conditions

**FIGURE 1:**
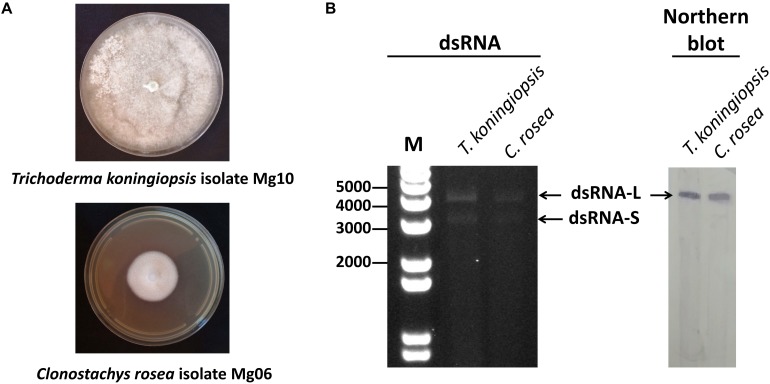
**(A)** Colony morphology of *Trichoderma koningiopsis* isolate Mg10 and *Clonostachys rosea* isolate Mg06 on potato dextrose agar (PDA). **(B)** The dsRNA profile (dsRNA-L and dsRNA-S) of *T. koningiopsis* isolate Mg10 and *C. rosea* isolate Mg06 on 1% (w/v) agarose gel (left panel) and northern hybridization detection of Trichoderma koningiopsis totivirus 1 (TkTV1/Mg10) represented by dsRNA-L in both fungal isolates (right panel) using DNA probes. M: 1 Kb Plus DNA Ladder (Invitrogen).

*Trichoderma koningiopsis* (isolate Mg10) and *C. rosea* (isolate Mg06) were isolated in 2016 from a single soil sample obtained from Mamaku, Rotorua using a standard isolation method (Warcup, 1950). Pure fungal cultures were obtained by single spore isolation and maintained and subcultured onto potato dextrose agar (PDA) plates (Figure 1A). Their identities were confirmed by amplifying and sequencing the non-coding internal transcribed spacer (ITS) regions using the primer pair ITS4/ITS5 (White et al., 1990). *T. koningiopsis* isolate TkF-T-11 and *C. rosea* isolate CrF-T-13 were produced by polyethylene glycol (PEG)-mediated mycovirus transfection of dsRNA-free isolates TkF and CrF of *T. koningiopsis* and *C. rosea*, respectively.

### Viral dsRNA and Fungal Total RNA Extraction

Viral dsRNA was extracted and purified from 400 mg of fungal mycelium using a method modified from that described by Valverde et al. (1990), as previously detailed by Khalifa and Pearson (2014). DsRNAs were resolved on 1% (w/v) agarose gel, in 1× TAE buffer (40 mM Tris, 20 mM glacial acetic acid, and 1 mM EDTA; pH 7.2) pre-stained with SYBR safe, and visualized and photographed under UV using a GelDoc (Bio-Rad). The dsRNA nature of the bands was confirmed by DNase and RNase treatments as described by Howitt et al. (1995). Total RNA was extracted from 100 mg of fungal mycelium, grown on cellophane over PDA medium for 5 days, using the Spectrum Plant Total RNA Kit (Sigma) as described by the manufacturer.

### Reverse Transcription-PCR and Sequencing

Purified dsRNAs were used as templates for RT-PCR using a protocol based on that of Roossinck et al. (2010) as described by Khalifa and Pearson (2013). RT-PCR products were purified using an Agencourt AMPure XP PCR purification kit (Beckman Coulter) and sequenced by Macrogen Inc. (Seoul, South Korea) using an Illumina HiSeq2000 platform. For sequence confirmation using Sanger sequencing, five pairs of dsRNA-L specific primers were designed and used to amplify overlapping fragments of dsRNA-L using a protocol described by Khalifa and Pearson (2013). The terminal sequences were amplified and sequenced as previously described by Khalifa and Pearson (2013). T4LC primer (5′-AAAGAGCCAGCAAACGACGGG-3′) was used as an adapter together with one of the specific primers 5′-ATTTTTGTCCGTGATGGG-3′ and 5′-TGTACTAGCATCGACGCC-3′ for the RT-PCR amplification of the 5′ and 3′ terminal sequences, respectively. RT-PCR products were cloned and sequenced by Macrogen Inc. (Seoul, South Korea). Bands corresponding to dsRNA-S from isolates Mg10 and Mg06 were excised separately and dsRNAs extracted using a gel extraction kit (AxyPrep). RT-PCR was performed and cDNAs partially sequenced to determine the identity of dsRNA-S as described by Khalifa and Pearson (2013).

### Northern Blot Analysis

A sequence-specific digoxigenin (DIG)-labeled probe was amplified using a PCR labeling kit (Roche), following the manufacturer’s protocol, with a primer pair of the following sequences: 5′-GTAAAGTAGGAGCCGTCC-3′ and 5′-TGTAAGAAGTACCAGTTCCC-3′. Northern blot hybridization and probe-RNA hybrid detection were performed as previously described by Khalifa and Pearson (2014).

### Bioinformatics, Sequence and Phylogenetic Analyses

Illumina reads with quality scores of less than Q20 were filtered out using The Galaxy Project server (Goecks et al., 2010), and low quality terminal sequence regions in the remaining reads were trimmed based on the FastQC report^1^. The remaining reads were assembled *de novo* using Geneious R8.1 (Kearse et al., 2012) set to medium sensitivity and default parameters. Encoded ORFs were detected using the ORF finder tool available at the National Center for Biotechnology Information (NCBI)^2^. Conserved motifs were determined by aligning corresponding sequences using PROMALS3D (Pei and Grishin, 2014).

For phylogenetic analyses, multiple sequence alignments of totiviral CP and RdRp aa sequences were performed using PROMALS3D (Pei and Grishin, 2014), and gaps were removed from the alignments using trimAl v1.3 (strict mode) (Capella-Gutierrez et al., 2009). Maximum likelihood phylogenetic trees were inferred using the selected best fitting-model of PhyML 3 (Guindon et al., 2010). Nodes with less than 75% SH-like branch support were collapsed using TreeGraph 2 software (Stöver and Müller, 2010).

### Purification of Virions and Protoplast Transfection

Fungal mycelium was grown on cellophane-covered PDA plates and 10 g of fungal mass was used for TkTV1 particles purification and transmission electron microscopic (TEM) visualization as previously described by Boine et al. (2012). TkTV1-free protoplasts were prepared from TkTV1-free isolates of *T. koningiopsis* (isolate TkF) and *C. rosea* (isolate CrF) using Glucanex and β-Glucuronidase as cell wall lytic enzymes (Sigma-Aldrich) as described by Shi-Wang et al. (2007) and Sun et al. (2017), respectively. For protoplast transfection, purified particles were passed through 0.45-μm syringe filters and used in PEG-mediated transfection tests of the previously prepared TkTV1-free protoplasts as described by Hillman et al. (2004). Transfected protoplasts were regenerated on regeneration medium for 4–5 days and 25 colonies for each fungus were randomly picked and transferred to PDA plates. DsRNA and total RNA were extracted as above and TkTV1 transmission was verified by RT-PCR amplification of an 879 bp-long fragment using specific primer pair TVF5 (5′-GTAAAGTAGGAGCCGTCC-3′) and TVR6 (5′-CTTCCAATTCGAGTGTTTCC-3′).

## Results and Discussion

With the advent of next-generation sequencing (NGS), mycovirus discovery from different sources has become more feasible (Ho and Tzanetakis, 2014). Mycoviruses from fungal material, environmental samples, insects, and other sources have been sequenced from dsRNA, total RNA and even siRNAs using NGS technologies (Al Rwahnih et al., 2011; Wu et al., 2012; Cook et al., 2013; Kraberger et al., 2013; Bruenn et al., 2015; Khalifa et al., 2016; Nerva et al., 2016). NGS platforms have been developed to produce nearly full-length reliable sequences that are comparable to those produced by conventional methods (Khalifa et al., 2016). In this study, NGS was utilized to sequence and identify a novel totivirus associated with two different fungal genera from dsRNA. The sequence was confirmed and the terminal sequences obtained using Sanger sequencing. The sequence was deposited in GenBank with the accession number MK993478.

### Fungal Identification and dsRNA Profiling

The nucleotide sequence of ITS region from isolates Mg10 and Mg06 shared the best match with publically available sequences of *T. koningiopsis* isolate TE26 (98.5% sequence identity; accession no. MH549097) and *C. rosea* isolate F6UP (98% sequence identity; accession no. MN252116), respectively.

Although nucleic acid bands of the same size do not necessarily have identical nucleotide sequences or represent the same virus, the banding patterns of dsRNAs purified from *T. koningiopsis* isolate Mg10 and *C. rosea* isolate Mg06 were nearly identical (Figure 1B). Both isolates contained two dsRNA segments that resisted digestions by DNase and RNase in high salt buffer, confirming their identity as dsRNAs. The estimated molecular weights of the two dsRNA segments were ∼5 kbp (dsRNA-L) and ∼3.5 kbp (dsRNA-S).

### Sequencing of dsRNA-L Segments

Illumina sequencing resulted in 583,356 and 173,454 short reads from *T. koningiopsis* isolate Mg10 and *C. rosea* isolate Mg06, respectively. Short Illumina reads were trimmed and quality filtered prior to being assembled using the Geneious 11.0.3 *de novo* assembly tool set to medium sensitivity and default parameters. Assembly of filtered reads corresponding to *T. koningiopsis* isolate Mg10 and *C. rosea* isolate Mg06 dsRNAs resulted in 31 and 20 contigs, respectively (only four contigs from each isolate were longer than 1 kbp). The longest contig obtained from isolate Mg10 dataset was 4052 nt long (367,651 assembled reads) and shared nearly the same nucleotide sequence with the longest contig (4477 nt; 82,871 assembled reads) obtained from isolate Mg06. Specific DNA hybridization probes were designed and northern blotting analysis revealed that the sequences of the two contigs from both isolates correspond to dsRNA-L (Figure 1B). RACE-PCR and sequencing of dsRNA-L terminal sequences resulted in a full-length sequence of dsRNA-L.

### Sequence Analysis of dsRNA-L

**FIGURE 2:**
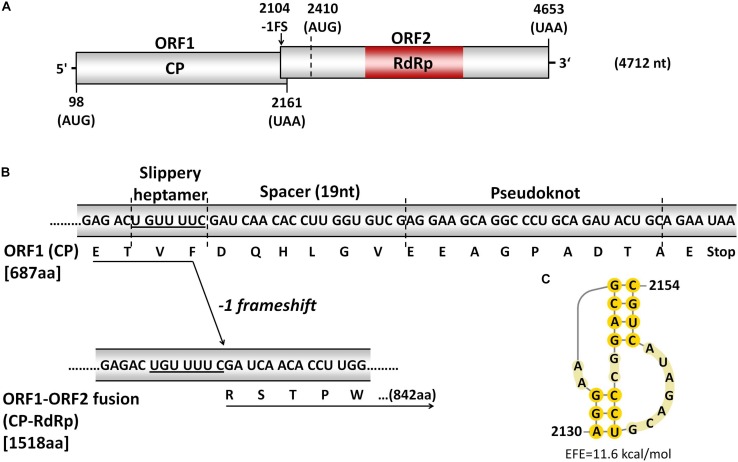
**(A)** Schematic representation of dsRNA-L (TkTV1/Mg10; 4712 nts). The coding strand contains two overlapping open reading frames (ORFs), indicated by boxes, and is flanked by 5′- and 3′-untranslated regions (UTRs) indicated by short lines. The putative slippery site for -1 ribosomal frameshifting is indicated by the black arrow. **(B)** The three genetic elements involved in -1 ribosomal frameshifting mechanism to produce ORF1-ORF2 fusion protein. **(C)** The predicted H-type pseudoknot secondary structure downstream of the potential slippery heptamer and spacer. EFE (kcal/mol) indicates the estimated free energy.

The complete Sanger sequencing confirmed sequence of dsRNA-L is 4712 nt long and contains two non-overlapping ORFs in reading frames 2 (ORF1) and 1 (ORF2) of its positive-sense strand, separated by a 248 nt long intergenic region, as revealed by ORF finder. Untranslated regions (UTRs) of 97 and 59 nt were detected at the 5′ and 3′ termini, respectively. ORF1 and ORF2 have the potential to code for 687 and 747 aa long proteins with predicted molecular weights of 77.652 and 85.114 kDa, respectively (Figure 2A). BLASTn search returned a single hit with 87% nt identity to a partial sequence of Snodland virus isolate UK1 (accession no. MF893257; E value: 0.0) associated with *Drosophila suzukii* (Medd et al., 2018). BLASTx searches of dsRNA-L nt sequence against NCBI databases revealed homologies to totiviruses and consequently, dsRNA-L from *T. koningiopsis* isolate Mg10 was tentatively named Trichoderma koningiopsis totivirus 1 (TkTV1/Mg10). The reason for proposing the TkTV1 name rather than Clonostachys rosea totivirus 1 (CrTV1/Mg06) is only because the dsRNA-L was detected and analyzed first in this isolate. It does not imply that the dsRNA has transmitted from *T. koningiopsis* to *C. rosea*. TkTv1/Mg06 genome shared the same length with that of TkTV1/Mg10 but differed in having five point mutations (99.89% sequence identity) at nt positions 84 (G to C; 5′-UTR), 1003 (C to T; ORF1), 1336 (G to A; ORF1), 1952 (G to C; ORF1), and 3507 (A to C; ORF2). Among the four substitutions located within the TkTV1 coding region, only one at nt position 1952 resulted in aa substitution, whereas the remaining three were silent.

The potential of TkTV1/Mg10 to code for two ORFs is typical for yeast totiviruses represented by Saccharomyces cerevisiae virus L-A (ScV-L-A) (Icho and Wickner, 1989; Kondo et al., 2016). ORF1 of TkTV1/Mg10 is 2064 nt long (nt positions 98-2161), starts at an AUG codon (nt positions 98-100) and terminates at an ochre (UAA) termination codon (nt positions 2159-2161). The length of ORF2 is 2244 nt, starting at nt positions 2410-4653 and it also terminates with a UAA codon, at nt positions 4651-4653 (Figure 2A).

Notably, the region preceding the stop codon of TkTV1/Mg10 ORF1 contains the three elements required to accomplish -1 ribosomal frameshifting in RNA viruses including totiviruses such as ScV-L-A (Dinman et al., 1991; Figure 2B). The first element is a slippery site, where frameshifting occurs, that consists of a shifty heptamer with the general sequence XXXYYYZ, where X represents A/G/C/U, Y represents A/U, and Z represents A/C/U (Bekaert and Rousset, 2005). The slippery site UGUUUUC was found at nt positions 2104-2110 of TkTv1/Mg10 genome. This corresponds to the slippery heptanucleotides found in ScV-L-A (GGGUUUA) (Brierley, 1995) and other related totiviruses, including *Xanthophyllomyces dendrorhous* viruses (GGAUUUU) (Baeza et al., 2012), *Puccinia striiformis* totiviruses (PsTVs) and red clover powdery mildew-associated totiviruses (RPaTVs; GG^G^/_A_UUUU) (Kondo et al., 2016; Zheng et al., 2017). The second element is a pseudoknot, downstream from the slippery site, that helps to pause translation and increase the rate of frameshifting (Rice et al., 1985). An H-type pseudoknot structure was detected at nt positions 2130-2154 of TkTV1/Mg10 genome (Figure 2C). The third element of the -1 frameshifting mechanism is a short spacer region between the slippery site and the pseudoknot, and is a 19 nt long stretch in TkTV1/Mg10. It was recently demonstrated that the presence of -1 ribosomal frameshifting elements does not necessarily lead to the expression of a fusion protein (Jamal et al., 2019). Although the expression of a CP-RdRp fusion protein by TkTV1 is yet to be confirmed experimentally, the presence of the -1 frameshifting signature in TkTV1 genome might induce the expression of an ORF1-ORF2 (CP-RdRp) fusion protein of 1518 aa with a molecular weight of 172.020 kDa. If this is the case, ORF2 devoid of any in-frame stop codons could possibly begin at nt positions 1990–1992 and the AUG start codon of ORF2 (nt positions 2410-2412) code for an internal methionine, resulting in a 172 nt long overlap between ORF1 and ORF2.

**FIGURE 3:**
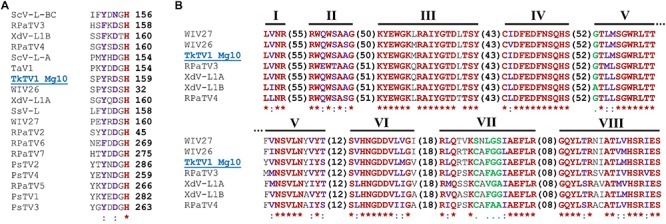
**(A)** A region extracted from the multiple amino acid (aa) sequence alignment of TkTV1/Mg10 coat protein (CP) and those of other totiviruses showing the position of the Histidine (His) aa residue required for the cap-snatching mechanism of totiviral CPs. **(B)** Conserved aa sequence motifs (I–VIII) of RNA-dependent RNA polymerase (RdRp) of totiviruses (members of group I, subgroup C are shown). Virus notations are as in Supplementary Table S1. Identical residues are marked in red and indicated by asterisks “^∗^”. Higher and lower chemically similar residues are marked in purple and green and signified by colons “:” and dots “.”, respectively.

ORF1 and ORF2 both code for viral proteins identified as CP and RdRp, using BLASTp searches, respectively. ORF1 protein shared aa sequence identities of 24–47% with hypothetical CPs of toti and toti-like viruses. TkTV1/Mg10 CP was most closely related to Wuhan insect virus 26 (WIV26; accession number YP_009342427, E-value 0.0, identity 47%), WIV27 (accession number YP_009342433, E-value 4e-156, identity 38%) and red clover powdery mildew-associated totivirus 4 (accession number BAT62483, E-value 4e-131, identity 35%). Multiple aa sequence alignment of TkTV1/Mg10 CP and those of other totiviruses revealed the presence of the aa residue required for the cap-snatching mechanism of totiviral CPs. In TkTV1/Mg10, this is represented by His-159 (Figure 3A) which corresponds to His-154 of ScV-L-A (Fujimura and Esteban, 2011), His-156 of ScV-L-BC (Fujimura and Esteban, 2013), and related aa residues in RPaTVs (Kondo et al., 2016). The prediction of a CP gene in TkTV1/Mg10 suggests that its genome is encapsidated, as for other totiviruses.

ORF2 protein showed aa sequence identities ranging from 26 to 57% with hypothetical RdRps of toti and toti-like viruses. TkTV1/Mg10 RdRp shared the highest aa sequence identities with WIV26 (accession number YP_009342428, E-value 0.0, identity 57%), WIV27 (accession number YP_009342434, E-value 0.0, identity 55%) and Delisea pulchra totivirus IndA (accession number AMB17470, E-value 0.0, identity 48%). Searching the conserved domain database (CDD) and multiple RdRp alignment confirmed the presence of a conserved viral RdRp domain (RdRp_4; pfam02123) with the eight conserved motifs (I–VIII) characteristic of the RdRps in dsRNA viruses (Routhier and Bruenn, 1998), including totiviruses, in the predicted RdRp sequence of TkTV1/Mg10 (Figure 3B).

### Identity of dsRNA-S

The remaining non-dsRNA-L-derived contigs were identified using BLASTx analysis with an E-value cut-off of 1 × 10^–6^. A single short virus-like contig was identified from *C. rosea* and shared similarities with Ditton virus (AWA82278), a negative-strand virus associated with *D. Suzukii*. Unlike dsRNA-L, Sanger sequencing of dsRNA-S and BLASTx results showed that the nucleotide sequence of dsRNA-S from isolates Mg10 and Mg06 were not similar. An 834 nt long fragment of dsRNA-S-Mg10 shared 47% aa identity with RdRp of Magnaporthe oryzae ourmia-like virus 4 (MK507958), whereas the partial sequence obtained for dsRNA-S from *C. rosea* (1107 nt) shared 59% aa sequence identity with RdRp of Botrytis cinerea mitovirus 4 (NC_028474). This might indicate that isolates Mg10 and Mg06 contained dsRNA-S prior to dsRNA-L cross-transmission. The full-characterization of dsRNA-S was not included in this study and will be described elsewhere in future studies.

### Phylogeny of TkTV1/Mg10

**FIGURE 4:**
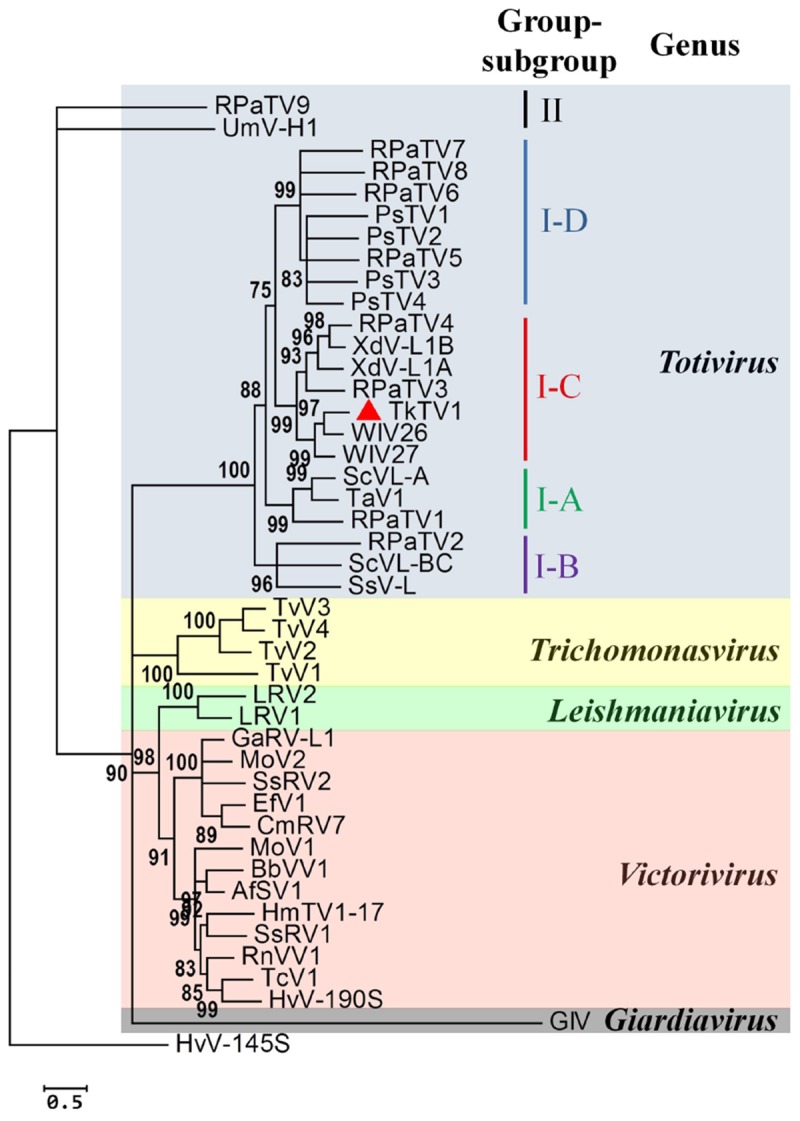
Phylogenetic relationship between the RNA-dependent RNA polymerase (RdRp) of Trichoderma koningiopsis totivirus 1 (TkTV1/Mg10) and other selected *Totiviridae* members. The maximum likelihood tree was inferred using PhyML 3.0 (Guindon et al., 2010) with the LG + G + I + F as the best evolutionary model. The SH-like support values are indicated by numbers on the branches. Branches with <75% SH-like branch support have been collapsed using TreeGraph 2 software (Stöver and Müller, 2010). Virus notations are as in Supplementary Table S1.

The current phylogeny status of *Totiviridae* classifies the family into five evolutionary lineages (genera), of which *Totivirus* is phylogenetically separated into two groups, I and II. Members of group I are further divided into four subgroups, A to D (Kondo et al., 2016). As shown in Figure 4 and Supplementary Figure S1, phylogenetic analyses based on CP and RdRp multiple alignments of TkTV1/Mg10 and corresponding toti and toti-like sequences revealed that TkTV1/Mg10 is most closely related to, but distinct from, members of group I, sub-group C (I-C). The ML trees inferred based on RdRp and CP had similar topologies in which TkTV1/Mg10 clustered in a sub-clade with WIV26 and WIV27 identified in flea and ants (Shi et al., 2016). Other members of sub-group C were isolated from a basidiomycetous fungus, *X. dendrorhous*, and the red clover powdery mildew ascomycete (Baeza et al., 2012; Kondo et al., 2016) suggesting possible horizontal virus transmission across different fungal phyla (Kondo et al., 2016). Moreover, it was suggested that uncharacterized totiviruses may infect a wide range of organisms across the different eukaryotic kingdoms such as fungi, plants, insects and red algae (Kondo et al., 2016), which is supported by the presence of closely related insect (WIV26 and WIV27) and fungal (TkTV1) totiviruses in a single sub-clade (Figure 4). As for Sclerotinia sclerotiorum hypovirulence-associated DNA virus 1 (SsHADV-1) which has the ability to infect a mycophagous insect that acts as transmission vector (Liu et al., 2016), transmission of TkTV1, WIV26, WIV27 and their ancestors may have been facilitated by mycophagous insects.

### Infectivity of TkTV1 Particles Using Transfection Assay

**FIGURE 5:**
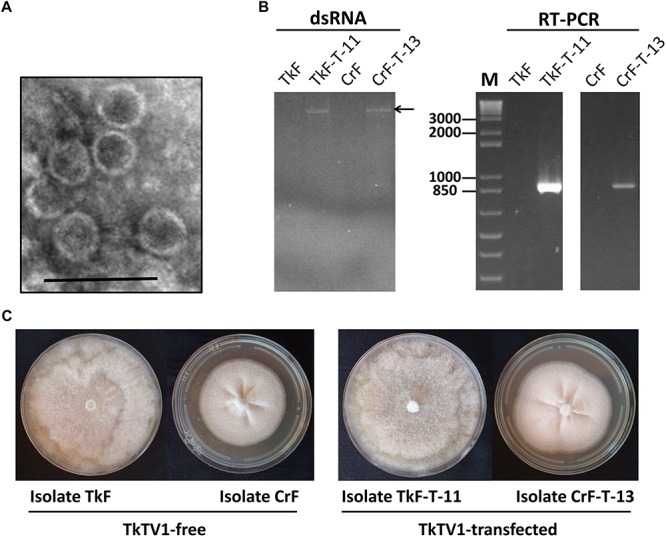
**(A)** Electron micrograph of negatively stained purified particles of Trichoderma koningiopsis totivirus 1 (TkTV1:38–40 nm in diameter). The Scale bar denotes 100 nm. **(B)** Detection of TkTV1 in TkTV1-free and TkTV1-transfected isolates of *T. koningiopsis* and *Clonostachys rosea*, using dsRNA extraction and RT-PCR. **(C)** Colony morphology of TkTV1-free and TkTV1-transfected isolates of *T. koningiopsis* and *C. rosea* grown potato dextrose agar (PDA) for 7 days.

Members of *Totiviridae* family have icosahedral virions that are 30–40 nm in diameter (Ghabrial et al., 2015). TkTV1 was purified and TEM examination showed that it has icosahedral virions of ∼38–40 nm in diameter (Figure 5A). TkTV1-free protoplasts of *T. koningiopsis* and *C. rosea* were transfected with TkTV1 virions purified from *T. Koningiopsis* isolate Mg10. Regenerated colonies of *T. koningiopsis* and *C. rosea* were nominated TkF-T-1 to -25 and CrF-T-1 to -25, respectively. Nine *T. koningiopsis* and two *C. rosea* colonies were tested positive for TkTV1 (Figure 5B) and showed identical colony morphology to those of original TkTV1-positive and TkTV1-free isolates (Figure 5C). Detection of TkTV1 in regenerated colonies confirms its ability to replicate in both fungal hosts.

### Implication of TkTV1 Presence in Different Hosts

Although the external route of RNA mycovirus transmission is still lacking, several studies have shed light on possible higher transmission rates of RNA mycoviruses in nature compared with their transmission *in vitro* in laboratory experiments. For instance, Cryphonectria hypovirus 1 (CHV1) was found to transmit between different species of a *Cryphonectria* natural population (Liu et al., 2003). Moreover, *in situ* transmission experiments of CHV1 between virus-infected and virus-free incompatible types revealed that the natural ability of CHV1 transmission between vegetatively incompatible groups was underrated by the results obtained from using pairing-culture technique (Brusini and Robin, 2013). Sclerotinia sclerotiorum mycoreovirus 4 (SsMYRV4) infection was reported to suppress host non-self recognition and facilitate heterologous horizontal virus transmission between SsMYRV4-infected and -uninfected incompatible fungal individuals (Wu et al., 2017). Also, previous studies reported the presence of highly similar mycoviral sequences in different fungal hosts, suggesting possible mycovirus transmission between different fungi, e.g., the presence of 92.4% nt and 95.1% aa identical sequences in *Sclerotinia homoeocarpa* and *Ophiostoma novo-ulmi* (Deng et al., 2003). Moreover, Entoleuca hypovirus 1 (EnHV1) was reported to infect *Entoleuca* sp. and *Rosellinia necatrix* (Velasco et al., 2018), and Botrytis porri RNA virus 1 was discovered in *S. sclerotiorum* (Mu et al., 2018) suggesting intra- and interspecific virus transmission. The presence of nearly identical sequences represented by TkTV1 in two different fungal genera provides clear-cut supporting evidence to previous reports suggesting the existence of lateral mycovirus transmission across different fungi in nature. The nearly identical sequence of TkTV1 in *T. koningiopsis* and *C. rosea* indicates that this transmission event has happened very recently in nature or probably while cultures were mixed prior to, or during, the isolation process.

## Data Availability Statement

TkTV1/Mg10 genome sequence is available in GenBank under the accession number MK993478.

## Author Contributions

Both authors secured the funding. MK undertook the research in consultation with RM and drafted the manuscript. RM revised the manuscript.

## Conflict of Interest

The authors declare that the research was conducted in the absence of any commercial or financial relationships that could be construed as a potential conflict of interest.

## Footnotes

1 http://www.bioinformatics.babraham.ac.uk/projects/fastqc/

2 http://www.ncbi.nlm.nih.gov/projects/gorf

## Supplementary Material

FIGURE S1 Phylogenetic relationship between the coat protein (CP) of Trichoderma koningiopsis totivirus 1 (TkTV1/Mg10) and other selected *Totiviridae* members. The maximum likelihood tree was inferred using PhyML 3.0 (Guindon et al., 2010) with the RtREV + G + F as the best evolutionary model. The SH-like support values are indicated by numbers on the branches. Branches with <75% SH-like branch support have been collapsed using TreeGraph 2 software (Stöver and Müller, 2010). Virus notations are as in Supplementary Table S1.

TABLE S1 Virus names and acronyms and GenBank accession numbers of proteins used to infer the phylogenetic trees.
