# Supplementary figures and images for: A Novel Totivirus Naturally Occurring in Two Different Fungal Genera
Source: Front Microbiol. 2019 Oct 11;10:2318. doi: 10.3389/fmicb.2019.02318 (PMC6797558; doi:10.3389/fmicb.2019.02318)

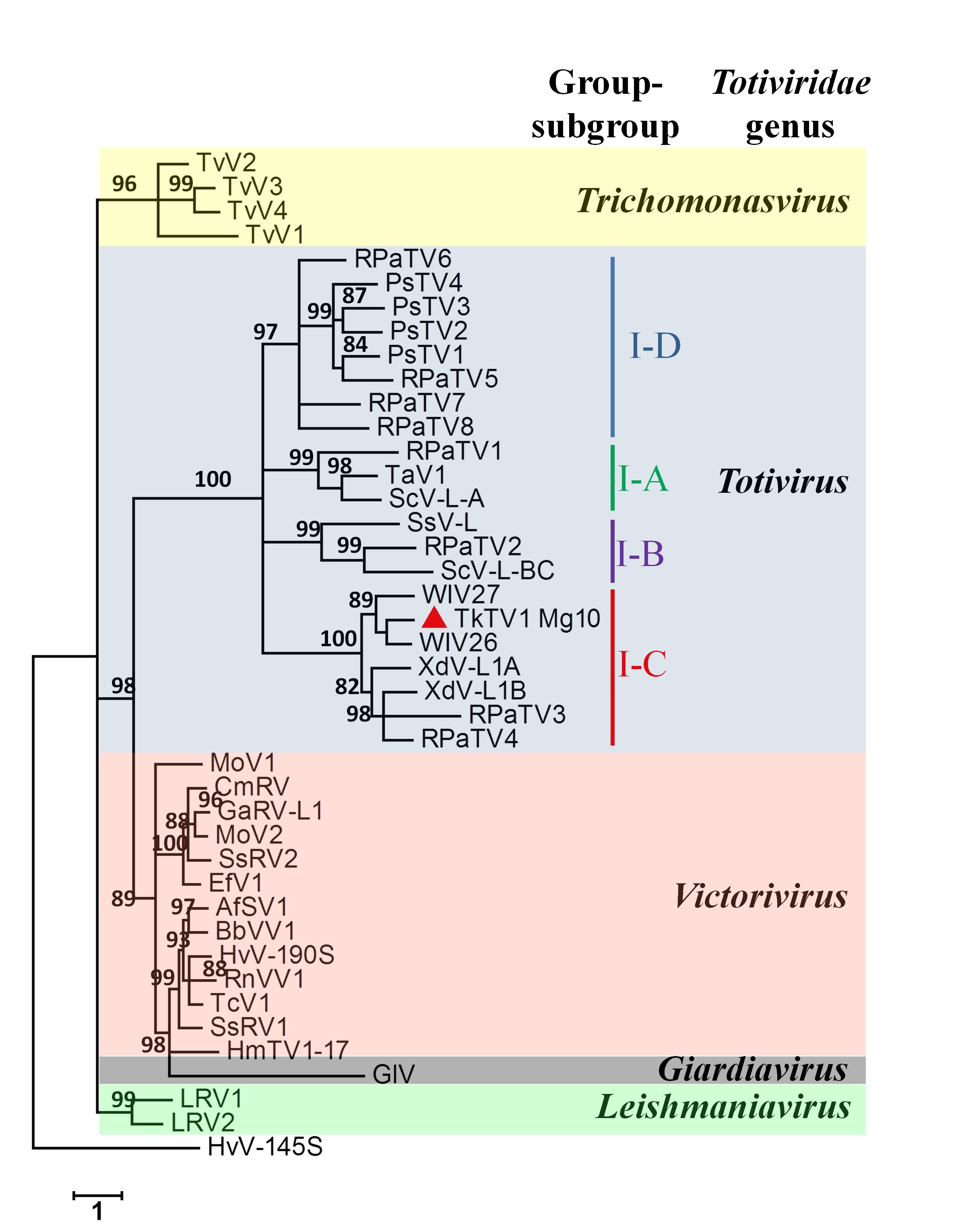

Supplement: FIGURE S1 — Phylogenetic relationship between the coat protein (CP) of Trichoderma koningiopsis totivirus 1 (TkTV1/Mg10) and other selected Totiviridae members. The maximum likelihood tree was inferred using PhyML 3.0 (Guindon et al., 2010) with the RtREV + G + F as the best evolutionary model. The SH-like support values are indicated by numbers on the branches. Branches with <75% SH-like branch support have been collapsed using TreeGraph 2 software (Stöver and Müller, 2010). Virus notations are as in Supplementary Table S1. [file Image_1.JPEG]
