# Supplementary material for: A Novel Totivirus Naturally Occurring in Two Different Fungal Genera
Source: Front Microbiol. 2019 Oct 11;10:2318. doi: 10.3389/fmicb.2019.02318 (PMC6797558; doi:10.3389/fmicb.2019.02318)
Supplement: TABLE S1 — Virus names and acronyms and GenBank accession numbers of proteins used to infer the phylogenetic trees. [file Table_1.docx]

**[Supplementary Table S1](https://www.frontiersin.org/articles/10.3389/fmicb.2019.00626/full#SM1):** Virus names and acronyms and GenBank accession numbers of proteins used to infer the phylogenetic trees.

| **Virus** | **Acronym** | **GenBank accession no.** | |
| --- | --- | --- | --- |
|  |  | **RdRp** | **CP** |
| ***Totivirus*** | | | |
| Puccinia striiformis totivirus 1 | PsTV1 | ATO91007 | ATO91006 |
| Puccinia striiformis totivirus 2 | PsTV2 | ATO91009 | ATO91008 |
| Puccinia striiformis totivirus 3 | PsTV3 | ATO91011 | ATO91010 |
| Puccinia striiformis totivirus 4 | PsTV4 | ATO91013 | ATO91012 |
| Red clover powdery mildew-associated totivirus 1 | RPaTV1 | BAT62476 | BAT62475 |
| Red clover powdery mildew-associated totivirus 2 | RPaTV2 | YP_009182176 | YP_009182175 |
| Red clover powdery mildew-associated totivirus 3 | RPaTV3 | BAT62482 | BAT62481 |
| Red clover powdery mildew-associated totivirus 4 | RPaTV4 | BAT62484 | BAT62483 |
| Red clover powdery mildew-associated totivirus 5 | RPaTV5 | BAT62486 | BAT62485 |
| Red clover powdery mildew-associated totivirus 6 | RPaTV6 | BAT62488 | BAT62487 |
| Red clover powdery mildew-associated totivirus 7 | RPaTV7 | BAT62490 | BAT62489 |
| Red clover powdery mildew-associated totivirus 8 | RPaTV8 | BAT62492 | BAT62491 |
| Saccharomyces cerevisiae virus L-A | ScV-L-A | NP_620495 | NP_620494 |
| Saccharomyces cerevisiae virus L-BC | ScV-L-BC | AAB02146 | AAB02145 |
| Scheffersomyces segobiensis virus L | SsV-L | AGG68771 | AGG68770 |
| Tuber aestivum virus 1 | TaV1 | YP_009507833 | YP_009507832 |
| Wuhan insect virus 26 | WIV26 | YP_009342428 | YP_009342427 |
| Wuhan insect virus 27 | WIV27 | YP_009342434 | YP_009342433 |
| Xanthophyllomyces dendrorhous virus L1A | XdV-L1A | YP_007697651 | YP_007697650 |
| Xanthophyllomyces dendrorhous virus L1B | XdV-L1B | YP_009507835 | YP_009507834 |
| Ustilago maydis virus | UmV-H1 | AAA81884 | |
| Red clover powdery mildew-associated totivirus 9 | RPaTV9 | YP_009182198 | |
| ***Victorivirus*** | | | |
| Aspergillus foetidus slow virus 1 | AfSV1 | YP_009508249 | YP_009508248 |
| Beauveria bassiana victorivirus 1 | BbVV1 | YP_009508251 | YP_009508250 |
| Coniothyrium minitans RNA virus | CmRV | YP_392467 | YP_392466 |
| Epichloe festucae virus 1 | EfV1 | YP_009508253 | YP_009508252 |
| Gremmeniella abietina RNA virus L1 | GaRV-L1 | NP_624332 | NP_624331 |
| Helicobasidium mompa totivirus 1-17 | HmTV1-17 | NP_898833 | NP_898832 |
| Helminthosporium victoriae virus 190S | HvV-190S | NP_619670 | NP_619669 |
| Magnaporthe oryzae virus 1 | MoV1 | YP_122352 | YP_122351 |
| Magnaporthe oryzae virus 2 | MoV2 | YP_001649206 | YP_001649205 |
| Rosellinia necatrix victorivirus 1 | RnVV1 | YP_008130308 | YP_008130307 |
| Sphaeropsis sapinea RNA virus 1 | SsRV1 | NP_047558 | NP_047557 |
| Sphaeropsis sapinea RNA virus 2 | SsRV2 | NP_047560 | NP_047559 |
| Tolypocladium cylindrosporum virus 1 | TcV1 | YP_004089630 | YP_004089629 |
| ***Trichomonasvirus*** | | | |
| Trichomonas vaginalis virus 1 | TvV1 | YP_009162330 | YP_009162331 |
| Trichomonas vaginalis virus 2 | TvV2 | NP_624323 | NP_624322 |
| Trichomonas vaginalis virus 3 | TvV3 | NP_659390 | NP_659389 |
| Trichomonas vaginalis virus 4 | TvV4 | YP_009507836 | YP_009507837 |
| ***Leishmaniavirus*** | | | |
| Leishmania RNA virus 1 | LRV1 | APT68186 | APT68185 |
| Leishmania RNA virus 2 | LRV2 | AAB50031 | AAB50030 |
| ***Giardiavirus*** | | | |
| Giardia lamblia virus | GlV | NP_620070 | NP_619551 |
| ***Chrysoviridae; Chrysovirus* (Outgroup)** | | | |
| Helminthosporium victoriae 145S virus | HvV-145S | YP_052858 | YP_052859 |
